# Self-confidence through teaching: Gender differences in medical students’ self-assessment of dermatological skills and learning effects

**DOI:** 10.3205/zma001869

**Published:** 2026-09-15

**Authors:** Ines Heinen, Julian Kött, Inga Hansen-Abeck, Rachel Sommer, Christine Blome, Isabel Heidrich, Martin Härter, Matthias Augustin, Stefan W. Schneider, Nina Booken, Finn Abeck

**Affiliations:** 1 University Medical Center Hamburg-Eppendorf, Department of Medical Psychology, Hamburg, Germany; 2 University Medical Center Hamburg-Eppendorf, Department of Dermatology and Venereology, Hamburg, Germany; 3 University Medical Center Hamburg-Eppendorf, Institute for Health Services Research in Dermatology and the Nursing Professions, Hamburg, Germany

**Keywords:** self-confidence, gender differences, self-assessment, exam performance, dermatological competencies, visual diagnosis

## Abstract

**Background:**

The ability to assess oneself realistically is central to medical education, as it enables students to identify their own strengths and weaknesses and can help prevent poor decision-making. Previous studies have shown that female students tend to underestimate their competencies, while male students tend to assess their abilities more realistically or overestimate them. The aim of the present study was to investigate gender-specific differences in the self-assessment of dermatological knowledge and skills among medical students and to compare these with objective test results.

**Methodology:**

As part of a seminar on skin diseases in Skin of Color (SoC) at the University Medical Center Hamburg-Eppendorf, a total of 142 students (57.7% female) participated in a quasi-experimental pre-post questionnaire survey during the winter semester of 2024-25. In addition to a multiple-choice (MC) test on eight dermatological visual diagnoses, self-assessments were collected on a 6-point Likert scale before and after the seminar.

**Results:**

At the outset, the female students rated their knowledge of anatomical and physiological differences between skin types (*p*<0.001, η^2^=0.078) and of scales used to determine skin types (*p*<0.001, η^2^=0.085), as well as their diagnostic skills regarding skin diseases in SoC, significantly worse than the male students (*p*<0.001, η^2^=0.075), although no differences were observed in the objective MC test (*p*=0.905). By the end of the seminar, no gender-specific differences remained.

**Conclusion:**

The results suggest that targeted instruction and feedback can promote self-confidence and realistic self-assessment, particularly among female students.

## Zusammenfassung

**Hintergrund:**

Die Fähigkeit zur realistischen Selbsteinschätzung ist zentral für die medizinische Ausbildung, da sie die Identifikation eigener Stärken und Schwächen ermöglichen und Fehlentscheidungen vorbeugen kann. Frühere Studien zeigten, dass Studentinnen ihre Kompetenzen tendenziell unterschätzen, während Studenten ihre Fähigkeiten eher realistisch bewerten oder zur Überschätzung neigen. Ziel der vorliegenden Studie war es, geschlechtsspezifische Unterschiede in der Selbsteinschätzung dermatologischer Kenntnisse und Fähigkeiten bei Medizinstudierenden zu untersuchen und diese mit objektiven Testergebnissen zu vergleichen.

**Methodik:**

Im Rahmen eines Seminars zu Hauterkrankungen bei Skin of Color (SoC) am Universitätsklinikum Hamburg-Eppendorf nahmen im Wintersemester 2024-25 insgesamt 142 Studierende (57,7% weiblich) an einer quasi-experimentellen Prä-Post-Fragebogenerhebung teil. Neben einem Multiple-Choice-Test (MC-Test) zu acht dermatologischen Blickdiagnosen wurden vor und nach dem Seminar Selbsteinschätzungen auf einer 6-stufigen Likert-Skala erhoben.

**Ergebnisse:**

Zu Beginn bewerteten die Studentinnen ihr Wissen über anatomische und physiologische Unterschiede zwischen Hauttypen (*p*<0,001, η^2^=0,078) und über Skalen zur Bestimmung von Hauttypen (*p*<0,001, η^2^=0,085) sowie ihre diagnostischen Fähigkeiten von Hauterkrankungen bei SoC signifikant schlechter als die Studenten (*p*<0,001, η^2^=0,075), obwohl sich in dem objektiven MC-Test keine Unterschiede zeigten (*p*=0,905). Am Ende des Seminars zeigten sich keine geschlechtsspezifischen Unterschiede mehr.

**Schlussfolgerung:**

Die Ergebnisse weisen darauf hin, dass gezielte Lehre und Feedback das Selbstvertrauen und die realistische Selbsteinschätzung insbesondere von Studentinnen

## Introduction

The ability to self-assess is an important component of medical education [1]. Being able to validly assess one’s own performance is important for both medical students and physicians, as this process enables the identification of individual strengths and weaknesses [2]. Overestimating one’s own competencies can increase the risk of clinical errors, while underestimation, combined with increased anxiety, can significantly impair performance [1], [3]. Therefore, continuous self-reflection in medicine is essential to ensure high-quality patient care [4]. The literature describes gender-specific differences in self-assessment among medical students: While women tend to underestimate their own abilities, men predominantly assess their competencies realistically or tend to overestimate them [1], [2], [5]. Previous studies have mostly been based on objective structured clinical examinations (OSCE) [1]. Gender differences were found, for example, in basic communication skills among first-semester students [6], in how students of all years interact with people with disabilities [7], and in psychiatry exams during the final year of study [8].

The aim of this study was to examine gender-specific differences in medical students’ self-assessment of their dermatological knowledge and diagnostic skills and to compare these with the objective results of a MC test on visual diagnoses.

## Methodology

students, took place between October 2024 and February 2025 at the University Medical Center Hamburg-Eppendorf (UKE) without a control group or randomization. The survey was administered during a 90-minute seminar on skin diseases in skin of color (SoC) [9], which was part of the regular curriculum in the 7^th^ semester of the integrated medical degree program iMED [10]. Due to the exhaustive sampling, an a priori power analysis was not performed. The study was approved by the local psychological ethics committee at the UKE (LPEK-0688).

The students completed the questionnaire at the beginning and at the end of the seminar; it focused on the diagnosis of eight skin conditions in SoC: psoriasis vulgaris, atopic dermatitis, tinea, varicella, vitiligo, melasma, melanoma, and keloids. For each skin condition, students were shown a photo for 20 seconds. They were then asked to select the correct visual diagnosis from six options in a MC test. During the seminar, these skin conditions were explained using additional photos and case reports. Different photos were shown in the two MC tests and in the seminar.

The students also assessed their knowledge of anatomical and physiological differences between lighter and darker skin, Fitzpatrick skin type classification scale (types I–VI), and their ability to diagnose skin diseases in SoC. For this purpose, three self-formulated items were used on a Likert scale from 1 to 6 (knowledge/ability ranging from “very low” to “very high”). All students were informed of the study prior to the survey. Participation was voluntary, anonymous, and unpaid. Non-participation had no negative consequences. Only fully completed questionnaires were analyzed.

Data analyses (ANOVA and paired t-test) were performed using IBM SPSS Statistics Version 29. The α-level of 5% was set to *p*=0.005 following Bonferroni correction for multiple testing.

## Results

Of the 172 students enrolled in the semester, 151 attended the seminar (87.8%); 142 students (94.0%) participated in the survey, of whom 57.7% were female (*n*=82), and the mean age was 25.2±2.6 years (range 21-35). At the beginning of the seminar, female students rated their knowledge of anatomical and physiological differences among various skin types, as well as scales for skin type classification significantly lower than male students (anatomical/physiological differences: f: 1.93±0.90, m: 2.52±1.14, F(1, 140)=13.008, *p*<0.001, η^2^=0.078; skin type scales: f: 1.98±0.97, m: 2.65±1.26, F(1, 140)=11.839, *p*<0.001, η^2^=0.085). Female students also initially rated their own ability to diagnose skin diseases in SoC lower (f: 1.74±0.75, m: 2.23±0.98, F(1, 140)=8.300, *p*<0.001, η^2^=0.075). However, the first MC test revealed no significant difference between the genders (f: 4.37±1.65, m: 4.40±1.73, *p*=0.905). After the seminar, no significant differences were observed among the students, neither in the self-assessments nor in the MC test (see figure 1 (Fig. 1) and table 1 (Tab. 1)). From the first to the second MC test (pre: 4.38±1.68; post: 7.40±1.17), the students showed a significant increase in correct answers (M-Diff 3.02±0.14, 95% CI [2.75; 3.30], t(141)=-21.732, *p*<0.001, d=1.657) – with no gender differences (f: 3.16±1.67, m: 2.83±1.64, F(1, 140)=1.338, *p*=0.249). As expected, the psychometric analysis of the three self-formulated items yielded maximally acceptable Cronbach’s alpha values, as they assessed self-ratings of knowledge and skills and were thus highly diverse in content. As expected, the items showed higher internal reliability in the post-survey than in the pre-survey (see table 2 (Tab. 2)).

## Discussion

To the best of our knowledge, this is the first study to examine gender-specific differences in the self-assessment of dermatological skills in combination with MC tests among medical students.

At the beginning of the seminar, female students significantly underestimated their skills compared to male students, even though their objective performance was comparable. This pattern is consistent with previous studies [1], [2], [5], [11]. This discrepancy disappeared by the end of the seminar, as the women – possibly due to an increase in self-confidence and self-efficacy – rated their competencies similarly to the men. Previous studies have shown that female students exhibit lower self-efficacy scores under conditions of uncertainty – such as those presented here by the new topic of SoC and the first MC test [11]. Female students assessed their abilities more cautiously at the beginning, but actively engaging with dermatoses in the context of SoC increased their self-assessment. Instructors should be aware of the potential for gender-specific self-undervaluation. Other factors influencing the female students’ initially lower self-assessment are possible but were not captured here and should therefore be further investigated, e.g., which elements of instruction contribute to fostering self-confidence and a realistic self-assessment – particularly among women.

## Limitations

The quasi-experimental design without a control group does not allow for strictly causal conclusions. Since the students took a MC test on eight diseases twice, learning effects resulting from retesting cannot be ruled out. This is a single-center study conducted at the UKE, which limits its generalizability. Furthermore, self-assessment within the context of a seminar is not equivalent to subsequent competence in patient care. Although a short-term improvement in learning was demonstrated for visual diagnoses, the question of long-term retention of knowledge remains open. Variables such as anxiety or self-confidence were not assessed, but could have acted as confounders and should therefore be included in future studies.

## Authors’ ORCIDs

- Ines Heinen: [0000-0001-7361-4554]
- Julian Kött: [0000-0003-2073-3909]
- Inga Hansen-Abeck: [0009-0007-5579-4337]
- Rachel Sommer: [0000-0002-3080-497X]
- Christine Blome: [0000-0002-1163-1639]
- Isabel Heidrich: [0000-0001-6894-1913]
- Martin Härter: [0000-0001-7443-9890]
- Matthias Augustin: [0000-0002-4026-8728]
- Stefan W. Schneider: [0000-0002-4679-7137]
- Nina Booken: [0009-0005-7692-9240]
- Finn Abeck: [0000-0001-7823-0736]

## Competing interests

The authors declare that they have no competing interests. 

## Figures and Tables

**Table 1:**
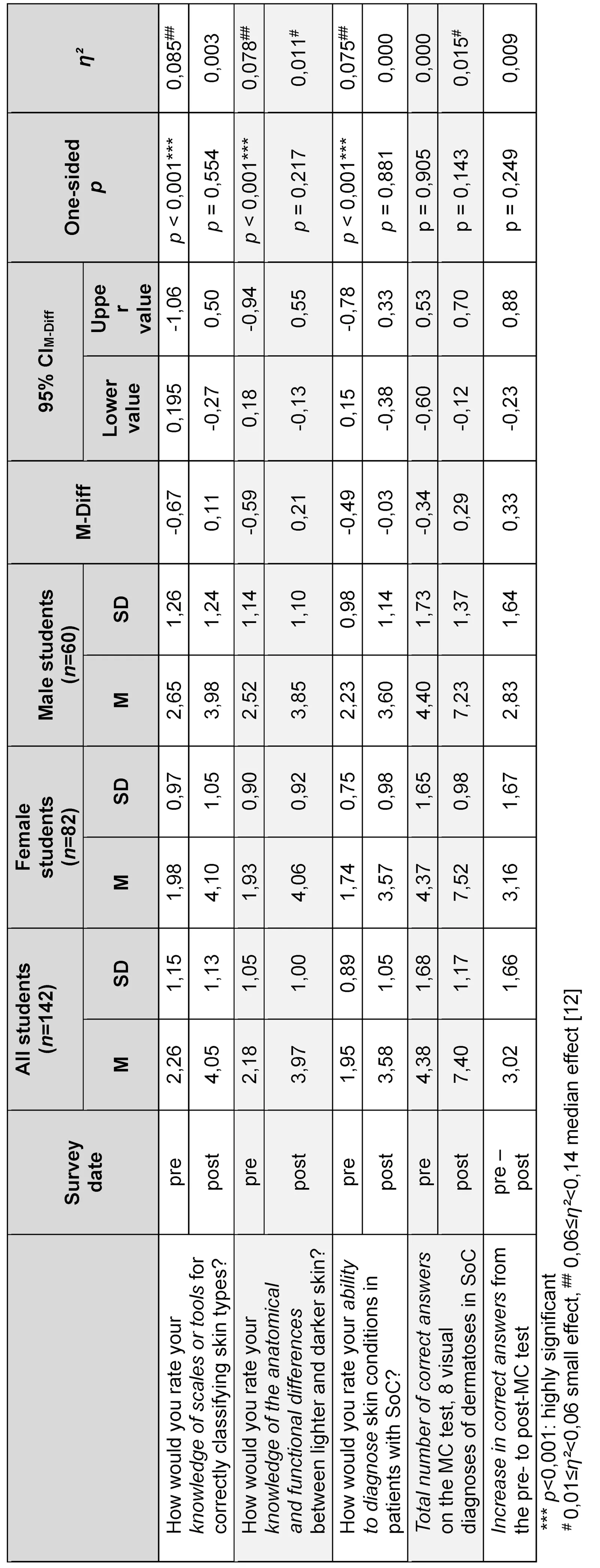
Descriptive statistics (M: mean, SD: standard deviation) for all students (n=142) and by gender. Results of the ANOVA with gender as group variable, including the mean difference (M-Diff) between groups, the 95% confidence interval for the mean difference (95% CI-Diff) and effect size Eta^2^ (η^2^) of the differences in self-assessments and results on the multiple-choice tests for female students (*n*=82) and male students (*n*=60) before (pre) and after (post) seminar participation. The last row shows the increase in correct answers from the 1^st^ to the 2^nd^ MC test, each with eight visual diagnoses of dermatoses in SoC.

**Table 2:**
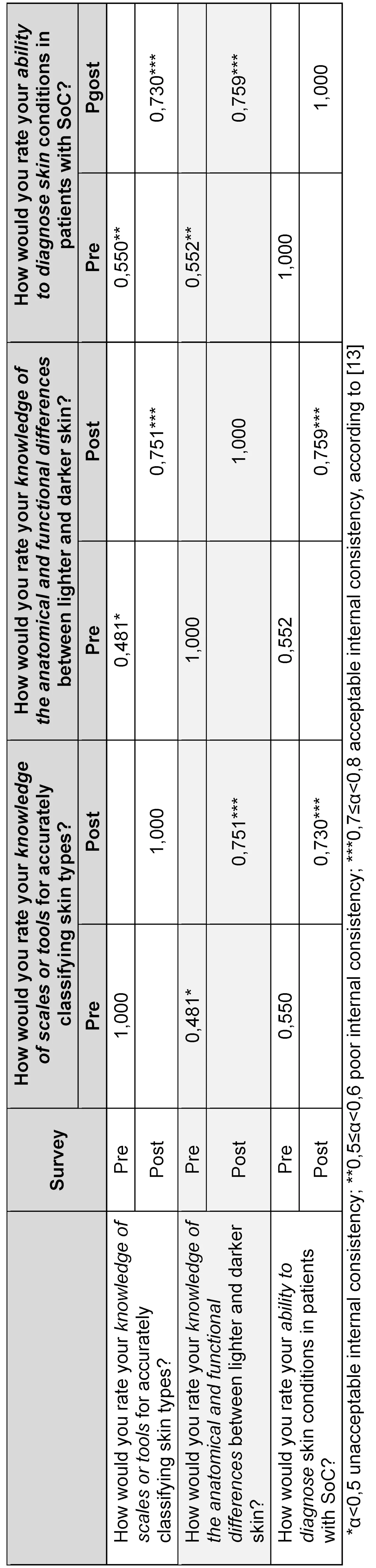
Results of the reliability analysis based on Cronbach’s alpha (α) for the three self-formulated items on self-assessed knowledge and self-assessed skills in the pre- and post-surveys (*n*=142 for each)

**Figure 1:**
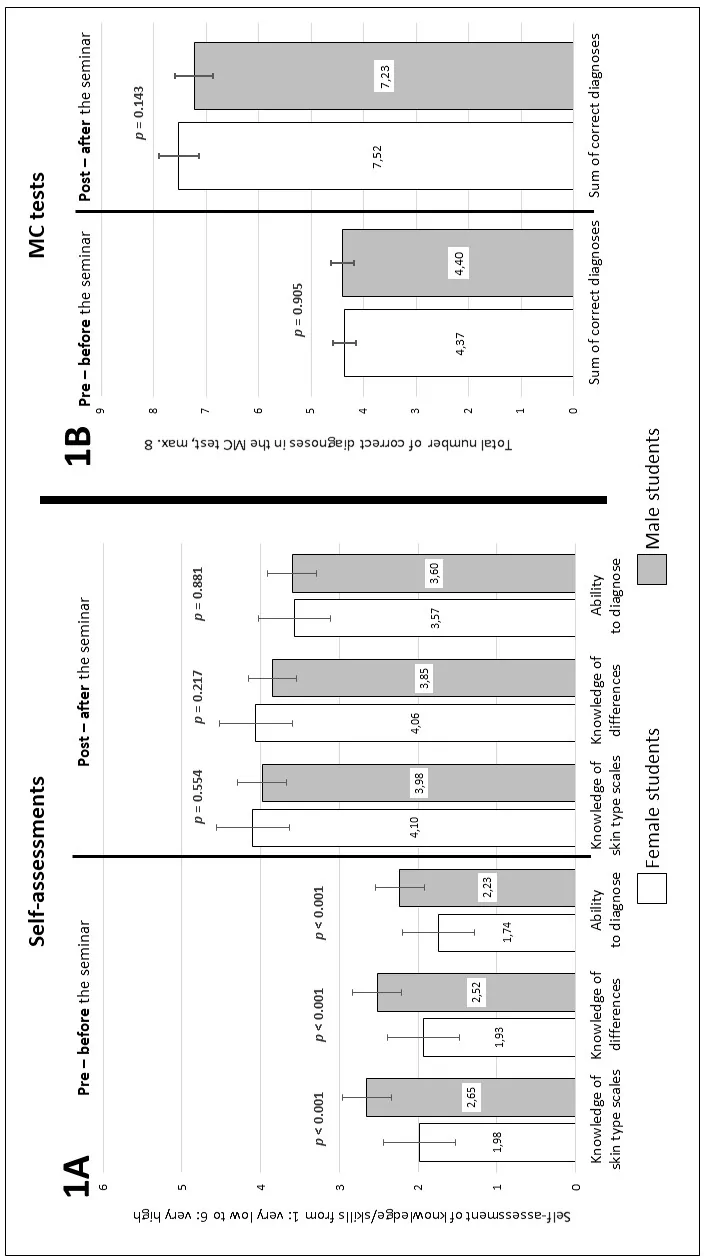
Results of the students (n=142, f=82, m=60) on the self-assessment questions and MC tests before (Pre) and after (Post) the seminar 1A: Self-assessments regarding knowledge of scales for classifying skin types (knowledge of skin type scales), knowledge of anatomical and physiological differences between lighter and darker skin (knowledge of differences), and the ability to diagnose skin diseases in SoC (ability to diagnose). 1B: Total number of correct diagnoses of dermatoses in SoC in the MC tests
